# Fertility Problem Inventory: Psychometric Properties and Validation in Spanish Women Undergoing Assisted Reproduction Treatment

**DOI:** 10.1007/s10880-026-10131-6

**Published:** 2026-02-12

**Authors:** Verónica Martínez-Borba, Jorge Osma, Jorge L. Ordóñez-Carrasco, Elena Crespo-Delgado, Laura Andreu-Pejó

**Affiliations:** 1 University of Zaragoza, Zaragoza, Spain; 2 Instituto de Investigación Sanitaria Aragón, Zaragoza, Spain; 3 Jaume I University, Castellón de la Plana, Spain

**Keywords:** Assisted reproduction treatments, Fertility problem inventory, Psychometric properties, Infertility-related stress, Spanish validation

## Abstract

The Fertility Problem Inventory (FPI) is widely used to assess infertility-related stress. Despite its international application, a validated Spanish version is not yet available. Our aim is to assess the psychometric properties, reliability and construct validity evidence of the scores of the FPI Spanish version in women undergoing fertility treatments. 199 Women (mean age = 35.14, *SD* = 3.86, range 23–45) provided sociodemographic, obstetrics and psychological data. Confirmatory Factor Analyses tested six models (unidimensional and multidimensional) based on the original FPI and previous FPI validations, identifying the optimal structure. Factor loadings, reliability and correlations with anxiety, depression and life satisfaction were also conducted. The original five-factor structure proposed by the original authors showed the best fit. However, items 24, 25 and 28 showed very low factor loadings. The final model with 43 items, removing those with poor factor loading, demonstrated an acceptable model fit (χ^2^/*df* = 2.040; CFI = 0.915; TLI = 0.910; RMSEA = 0.073; SRMR = 0.099) and strong internal consistency (α = 0.82–0.87; ω = 0.83–0.87). Higher fertility-related stress was associated with increased anxiety and depressive scores as well as lower life satisfaction. These findings provide initial support of the FPI as a useful tool for assessing infertility-related stress in Spanish women.

**Supplementary Information:**

The online version contains supplementary material available at https://doi.org/10.1007/s10880-026-10131-6.

## Introduction

Infertility is a major health problem affecting millions of women and men worldwide. Globally, 12.6–17.5% of people of reproductive age experience infertility (Cox et al., 2022), with similar prevalence (17.6%) in Spain (World Health Organization, WHO, 2023). When facing difficulties achieving pregnancy, many couples choose assisted reproduction treatment (ART). Its use has increased markedly worldwide in recent decades (Smeenk et al., 2023), with Spain being one of the most active countries in Europe. For example, in 2019, Europe averaged 1,077,813 cycles, while Spain accounted for 137,276 of them (Smeenk et al., 2023).

The evidence shows that ART can be considered highly stressful procedures since, in addition to carrying physical discomfort, they can have a negative impact on women's quality of life due to the constant uncertainty they generate, their long duration and the physical and emotional strain they entail (Assaysh-Öberg et al., 2023). Not surprisingly, some studies have shown that between 40 and 54% of women undergoing ART experienced stress, anxiety and depressive symptoms with high rates of comorbidity between conditions (Gupta et al., 2024; Kato et al., 2021; Lakatos et al., 2017; Zurlo et al., 2020).

In this scenario, international organizations such as the European Society of Human Reproduction and Embryology (ESHRE) have emphasized the importance of providing routine psychosocial assessments for women suffering infertility or undergoing ART (Gameiro et al., 2015), including anxiety, depression, quality of life and stress. Such assessments in medical settings may facilitate the correct and early identification of emotional suffering and the referral to the most convenient psychological program (prevention or treatment).

In view of this situation, over the last years, several instruments have been developed for the assessment of different aspects related to stress in people with infertility problems. For example, the Infertility Questionnaire (IFQ; Bernstein et al., 1985); the Fertility Problem Stress Inventory (FPS; Abbey et al., 1991); the Infertility Reaction Scale (IRS; Collins et al., 1992); or the Fertility Adjustment Scale (Glover et al., 1999). However, the most widely used infertility-related stress assessment tool in the research field is the Fertility Problem Inventory (FPI: Newton et al., 1999). The FPI has been translated into different languages (Portuguese, Greek, Italian, Mandarin, Kannada, and Iranian) and numerous FPI validation studies have been carried out in diverse populations of men and women with infertility (e.g., Donarelli et al., 2015; Moura-Ramos et al., 2012; Patel et al., 2022; Peng et al., 2011; Samani et al., 2017). The FPI is a multidimensional self-administered instrument that aims to identify infertility-related problems and it was originally developed as a global measure of stress that can also be used as a multidimensional scale to assess stress levels across five distinct domains: Social Concerns, Sexual Concerns, Relationship Concerns, Rejection of a Childfree Lifestyle, and Need for Parenthood (Newton et al., 1999). Some international validations of the FPI have corroborated its five-factor structure (e.g., Kim & Shin, 2014; Patel et al., 2022; Peng et al., 2011; Samani et al., 2017), however, validation studies conducted in Italy and Portugal (Donarelli et al., 2015; Moura-Ramos et al., 2012) found that the subscales of rejection of a childfree lifestyle and need for parenthood could be merged. Accordingly, they proposed a two-factor solution: one called “Impact on life domains” (which includes the original Social, Sexual and Relationship Concerns subscales) and the other called “Importance of parenthood” (encompassing the original Rejection of Childfree Lifestyle and Need for Parenthood subscales).

Although there is considerable interest in validating the FPI across different cultural contexts, and despite its usefulness for assessing stress in individuals undergoing fertility treatments (i.e., Galhardo et al., 2013; Moura-Ramos et al., 2017; Zurlo et al., 2020), to the best of our knowledge, a validated version of the FPI is not yet available for the Spanish population. Therefore, the aim of our study is to assess the psychometric properties, reliability and construct validity evidence of the scores of the Spanish version of the FPI in women undergoing ART.

## Method

### Sample

To participate in the study, women had to meet the following inclusion criteria: (1) to be at least 18 years of age; (2) to be undergoing ART; (3) to be fluent in Spanish, the language in which the evaluation was carried out, and (4) to sign the informed consent form. In addition, exclusion criteria were also included: (1) not having an electronic device to connect to Internet and be able to carry out the evaluation.

### Procedure

The research and all its procedures were approved by the relevant Ethics and Research Committees of the General University Hospital from Castellón and the University Hospital La Plana from Vila-Real.

All women who participated in this study received a flyer with information about the study. To reach as many participants as possible, the flyer was distributed via advertisement on social media (i.e., Instagram and Facebook) and it was also displayed in two human reproduction units in Spain (General University Hospital of Castellón and University Hospital La Plana of Vila-Real). The flyer included the basic information about the study as well as access to the online platform *Qualtrics* (participants could choose to scan a QR or click on an URL link). Once on the online platform *Qualtrics*, the study information and the informed consent were presented. After reading both documents, the participant voluntarily gave her written online consent to participate in the study on the same *Qualtrics* platform. Then, participants were presented with the assessment protocol, including the FPI and other psychosocial measures. The time required to complete the questionnaires was approximately 20–30 min. The evaluation process was conducted online without any involvement from healthcare professionals. Participation in the study was voluntary, anonymous and did not include any financial compensation.

### Measures

The *Fertility Problem Inventory* (FPI; Newton et al., 1999) is a 46-item, self-administered measure that divides infertility-related problems into five homogeneous domains: Social concern (items 1–10): “*It doesn’t bother me when I’m asked questions about children*” (item 10); Sexual concern (items 11–18): “*I find I’ve lost my enjoyment of sex because of the fertility problem*”(item 11); Relationship concern (items 19–28): “*I can’t show my partner how I feel because it will make him/her feel upset*”(item 19); Rejection of a childfree lifestyle (items 29–36): “*Couples without a child are just as happy as those with children*” (item 29); Need for parenthood (items 37–46): “*Pregnancy and childbirth are the two most important events in a couple’s relationship*” (item 37). Each item is rated according to the degree of agreement on a six-point Likert scale (from 1 = strongly disagree to 6 = strongly agree). Eighteen items were reverse coded (items: 1, 2, 6, 10, 12, 13, 21, 25, 28–36 and 43). The score for each subscale is obtained by adding the scores of the corresponding items. As proposed in the original FPI, an overall score can also be obtained by adding up the scores of all items. This score is interpreted as a global measure of perceived infertility-related stress. The overall score ranges from 46 to 276, where the higher the score, the greater the infertility-related stress.

The Spanish version of the FPI was obtained after translation into Spanish and a back translation into English to ensure conceptual equivalence. Following the International Test Commission recommendations (ITC, 2017), the translation into Spanish was carried out by two independent researchers who were proficient in English. These researchers compared and corrected discrepancies in the translations. This Spanish version was back translated into English by two independent English native translators who were fluent in Spanish. It should be noted that some general language adjustments were made. For instance, grammatical adjustments were made to certain items to refer not only to fertility problems but also to receiving ART (i.e., “My partner doesn’t understand the way the fertility problems/*treatments* affect me”; see attached instrument to see the final version of the questionnaire, Appendix A).

The *Overall Anxiety Severity and Impairment Scale* (OASIS; Norman et al., 2006; Osma et al., 2019) presents five items related to the frequency of anxiety symptoms during the last week, their intensity, their interference with the person's work or school life, and their interference with social life. All items are rated on a 5-point Likert scale ranging from 0 (I didn't feel anxious) to 4 (Constant anxiety). The total scale score ranges from 0 to 20 points and is obtained by adding up all item scores. Higher scores indicate greater severity and functional impairment (Norman et al., 2006). The cut-off point in a sample of Spanish people with emotional disorders cared for in the National Health System (NHS) was 10 (Osma et al., 2019). In the OASIS Spanish validation, Cronbach’s alpha was α = 0.87 (Osma et al., 2019) while in this study it was higher (α = 0.93).

The *Overall Depression Severity and Impairment Scale* (ODSIS; Bentley et al., 2014; Osma et al., 2019) includes five items related to the frequency of depressive symptoms, their intensity, the loss of interest or difficulty in participating in activities, their interference with the person's work or school life, and their interference with social life. Items in the ODSIS use a 5-point Likert response scale ranging from 0 (I didn't feel depressed) to 4 (Constant depression). Again, total scores range from 0 to 20 points after adding up all items, with higher values revealing a more severe and functional impairment as a result of depressive symptoms (Bentley et al., 2014). The cut-off point in a sample of Spanish people with emotional disorders cared for in the NHS was also 10 (Osma et al., 2019). In the ODSIS Spanish validation, Cronbach’s alpha was α = 0.94 (Osma et al., 2019), which is similar to the values obtained in our sample (α = 0.95).

The *Satisfaction With Life Scale* (SWLS; Diener et al., 1985; Vázquez et al., 2013) is a 5-item scale designed to measure global cognitive judgments of one’s life satisfaction where participants indicate how much they agree or disagree with each of them using a 7-point scale that ranges from 1 (I strongly disagree) to 7 (I strongly agree), and the higher the scores, the higher the life satisfaction. SWLS Cronbach’s alpha in the Spanish validation was α = 0.88 (Vázquez et al., 2013), which is comparable to the reliability coefficient obtained in our sample (α = 0.86).

### Statistical Analyses

First, a description of the sample was made using mean and standard deviation for the ordinal variables and frequencies and percentages for the nominal variables.

In order to examine the internal structure of the FPI, a confirmatory factor analysis (CFA) was performed with three alternative models proposed by previous FPI validations: a one-factor model with the 46 items (model 1); a multidimensional model with the five factors suggested by the original authors (model 2); a multidimensional model with two factors (model 3), one called impact on life domains (including the subscales of social, sexual and relationship concerns) and the other one called representations about the importance of parenthood (including rejection of childfree lifestyle and need for parenthood) proposed by Moura-Ramos et al. (2012) and Donarelli et al. (2015). Although an exploratory factor analysis (EFA) followed by CFA is often recommended in scale validation (Brown, 2015), this approach requires adequate sample size to ensure stable and replicable factor solutions. Given these constraints, we prioritized a CFA approach grounded in the theoretically specified structure of the instrument, rather than performing exploratory analyses that could lead to empirically driven but potentially unreliable model modifications (Kline, 2016).

The factor loadings of the items in each model were also assessed. Considering the recommendations in the literature (Kline, 2016) and the criteria applied in previous FPI validations (Peng et al., 2011) a minimum factor loading of 0.30 was required for an item to be retained in the model. Since three items obtained low factor loading (items 24, 25 and 28), the original structure of the FPI was further explored after removing these problematic items. Thus, three additional FPI models were tested: one based on a global measure of stress with 43 items (model 4); another using the original five-factor structure with 43 items (model 5) and a third following the two-factor structure with 43 items (model 6). The CFA were conducted using the Diagonally Weighted Least Squares (DWLS) estimation method, as it is recommended for ordinal and/or non-normal data. The fit of each model was assessed using the following indices: χ^2^/df (ratios of chi-square to degrees of freedom < 2 or < 3), CFI (> 0.90), RMSEA (< 0.05–0.08) and SRMR (< 0.08–0.10), following the criteria recommended in the literature (Kline, 2016).

Then, to assess the reliability of the scores on each dimension of the FPI, Cronbach’s Alpha coefficient and McDonald’s Omega coefficients were calculated.

To get the descriptive statistics of items and dimensions of the FPI, the mean, standard deviation, skewness and kurtosis of each item and the five dimensions were calculated with final factor solution (i.e., five-factor solution with 43 items).

Finally, Pearson correlation coefficients were computed to assess the factor correlations of the five-factors multidimensional model and the construct validity evidence based on the relationships between the FPI dimensions and the other measures (OASIS, ODSIS and SWLS). All the statistical analyses were performed with JASP (JASP Team, 2025).

## Results

### Sociodemographic Characteristics of the Sample

The sample comprised 199 women who agreed to participate in the study and completed all questionnaires. The total sample had a mean age of 35.14 years (*SD* = 3.86, range 23–45). The majority had a university degree (n = 123, 62.10%); a high or very high economic level (n = 127, 65.5%) and an active job (n = 157, 78.9%). Regarding obstetric variables, the majority of participants had no previous miscarriages (n = 112, 56.30%) and no previous children (n = 182, 91.50%). In most cases, the participants did not know the cause of the infertility problem (n = 83, 41.70%), were receiving ART in a public health center (n = 139, 69.80%) and usually had “in vitro fertilization” (IVF, n = 116, 58.60%). For psychological variables, the mean scores for anxiety and depression were 6.44 (*SD* = 4.96) and 6.06 (*SD* = 5.14) points respectively and the mean score for life satisfaction was 21.97 points (*SD* = 6.56). The scores for fertility-related stress ranged from 18.12 (*SD* = 7.70; relationship concern) to 37.85 (*SD* = 9.30; need for parenthood, see Table 1).

**Table 1:** Sociodemographic, obstetric and psychological characteristics of the sample

Variable	M (*SD*; range)
Age	35.14 (3.86; 23–45)
ART time (months)	13.48 (17.48; 1–121)
Depression (ODSIS)	6.06 (5.14; 0–20)
Anxiety (OASIS)	6.44 (4.96; 0–20)
Life satisfaction	21.97 (6.56; 5–34)

	N (%)
Previous miscarriages	
Yes	87 (43.60)
No	112 (56.30)
Academic level	
Primary school	5 (2.50)
Secondary school	15 (7.60)
High school	12 (6.10)
Professional training/education	43 (21.70)
University studies	123 (62.10)
Employment status	
Active	157 (78.9)
Temporary leave	18 (9.00)
Unemployed	23 (11.60)
Household work	1 (0.50)
Annual income	
< 15,000€	58 (29.9)
15,000–25,000€	69 (35.60)
25,000–35,000€	50 (25.80)
35,000–40,000€	9 (4.60)
> 40,000€	8 (4.10)
Children of their own	
0	182 (91.50)
1	15 (7.50)
2	2 (1.00)
Type of ART center	
Public	139 (69.80)
Private	57 (28.60)
Infertility cause	
Women	62 (31.20)
Partner	24 (12.10)
Both	30 (15.10)
Unknown	83 (41.70)
Type of ART	
Planned intercourse	4 (2.00)
AI	78 (39.40)
IVF	116 (58.60)

*M* Mean, *SD* Standard Deviation, *ODSIS* The Overall Depression Severity and Impairment Scale, *OASIS* The Overall Anxiety Severity and Impairment Scale, *FPI* Fertility Problem Inventory, *ART* Assisted Reproduction Treatments, *AI* Artificial Inseminations, *IVF* In Vitro Fertilization

### FPI Structure

As can be observed in Table 2, it was found that the one-factor solution (model 1) and the two-factor solution (impact on life domains and representations about the importance of parenthood; model 3) did not fit well in our sample, while the original multidimensional model with five factors had an acceptable fit (model 2).

**Table 2:** Goodness-of-fit Indices for the CFA Models

Models	χ^2^	*df*	χ^2^/*df*	CFI		RMSEA				SRMR
					TLI	Est.	90% CI		*p*	
							LL	UL		
Model 1 (unidimensional)	3953.752	989	3.998	0.718	0.705	0.124	0.120	0.128	< .001	0.141
Model 2 (multidimensional 5 original factors)	1894.450	979	1.935	0.913	0.908	0.069	0.064	0.074	< .001	0.097
Model 3 (multidimensional 2 factors)	2315.876	988	2.344	0.874	0.868	0.083	0.078	0.087	< .001	0.107
Model 4 (unidimensional, 43 items)	3791.856	860	4.409	0.719	0.705	0.132	0.127	0.136	< .001	0.148
Model 5 (multidimensional, 5 factors, 43 items)	1734.080	850	2.040	0.915	0.910	0.073	0.068	0.078	< .001	0.099
Model 6 (multidimensional 2 factors, 43 items)	2154.949	859	2.509	0.876	0.870	0.088	0.083	0.092	< .001	0.110

*Df* degrees of freedom, *CFI* Comparative fit index, *Est.* Estimate, *RMSEA* Root Mean Square Error of Approximation, *CI* Confidence interval, *LL* Lower limit, *UL* Upper limit, *SRMR* Standardized root mean square residual

The factor loadings of this original five-factor model were examined to determine whether the items showed adequate loadings on their respective factors (Table 3). The factor loadings were generally high, ranging from − 0.070 (item 25) to 0.851 (item 31), but three items (item 24 “*My partner and I could talk more openly with each other about our fertility problem/treatment*”, item 25 “*I couldn’t imagine us ever separating because of this*”, and item 28 “*When we talk about our fertility problem/treatment, my partner seems comforted by my comments*”) had very low loadings on their respective factor. The factor loadings for the five-factor model with 43 items were similar to the previous model, ranging from 0.301 (item 2) to 0.851 (item 31), but without the problematic items (see Table 3 for a detailed presentation of factor loadings for all items).

**Table 3:** Factor loadings

Factors	Items	Five-factors model	
		With 46 items	With 43 items
Social concern (SC)	FPI_1	0.452	0.451
	FPI_2	0.301	0.301
	FPI_3	0.661	0.660
	FPI_4	0.688	0.688
	FPI_5	0.743	0.742
	FPI_6	0.352	0.352
	FPI_7	0.669	0.669
	FPI_8	0.757	0.758
	FPI_9	0.672	0.665
	FPI_10	0.548	0.540
Sexual concern (SeC)	FPI_11	0.701	0.701
	FPI_12	0.569	0.568
	FPI_13	0.433	0.428
	FPI_14	0.624	0.624
	FPI_15	0.614	0.615
	FPI_16	0.689	0.689
	FPI_17	0.598	0.599
	FPI_18	0.720	0.720
Relationship concern (RC)	FPI_19	0.749	0.750
	FPI_20	0.695	0.695
	FPI_21	0.594	0.591
	FPI_22	0.647	0.646
	FPI_23	0.547	0.551
	FPI_24	** − 0.015**	–
	FPI_25	** − 0.070**	–
	FPI_26	0.731	0.733
	FPI_27	0.669	0.665
	FPI_28	**0.045**	–
Rejection of childfree lifestyle (RCL)	FPI_29	0.610	0.609
	FPI_30	0.765	0.758
	FPI_31	0.851	0.851
	FPI_32	0.489	0.490
	FPI_33	0.577	0.577
	FPI_34	0.839	0.839
	FPI_35	0.682	0.682
	FPI_36	0.607	0.607
Need for parenthood (NP)	FPI_37	0.414	0.414
	FPI_38	0.633	0.633
	FPI_39	0.670	0.669
	FPI_40	0.469	0.469
	FPI_41	0.765	0.765
	FPI_42	0.627	0.620
	FPI_43	0.542	0.543
	FPI_44	0.540	0.541
	FPI_45	0.466	0.466
	FPI_46	0.576	0.577

Factorial loads below .30 in bold

Based on the information provided by the CFA models and the factor loadings, the original unidimensional model, the five-factor solution and the two-factor solution, removing the problematic items were also explored. The original unidimensional solution with 43 items (model 4) and the two-factor solution with 43 items (model 6) demonstrated poor fit. The multidimensional five-factor solution model with 43 items had a comparable fit to the original five-factor solution with 46 items. Therefore, it was decided to retain the model of 43 items and five factors, as it was the one that presented the best balance between parsimony and fit to the data (see Table 2).

### Reliability

The results showed that all the dimensions had high internal consistency, with Alpha values ranging from 0.82 to 0.87 and McDonald’s omega values ranging from 0.83 to 0.87 (Social Concern, α = 0.84, ω = 0.84; Sexual Concern, α = 0.82, ω = 0.83; Relationship Concern, α = 0.84, ω = 0.85; Rejection of Childfree Lifestyle, α = 0.87, ω = 0.87; Need for Parenthood, α = 0.83, ω = 0.84).

### Descriptive Statistics of Items and Dimensions

As observed in Table 4, the means of the items range from 1.402 (item 23) to 4.975 (item 45), indicating a wide variability in the responses. Most of the items have means higher than the midpoint of the scale, suggesting a moderate or high level of agreement with the statements of the FPI.

**Table 4:** Descriptive statistics of each item and each dimension, as well as the total score of the scale

Items	Mean	SD	Skewness	Kurtosis
FPI_1	3.899	1.772	− 0.308	− 1.276
FPI_2	2.904	1.691	0.496	− 1.053
FPI_3	2.407	1.602	0.789	− 0.708
FPI_4	2.749	1.687	0.542	− 0.956
FPI_5	3.899	1.726	− 0.432	− 1.000
FPI_6	3.060	1.523	0.469	− 0.643
FPI_7	3.005	1.805	0.315	− 1.332
FPI_8	3.065	1.655	0.246	− 1.135
FPI_9	1.924	1.325	1.489	1.415
FPI_10	3.131	1.727	0.289	− 1.215
FPI_11	3.563	1.765	− 0.133	− 1.327
FPI_12	3.513	1.778	− 0.015	− 1.375
FPI_13	2.915	1.675	0.377	− 1.211
FPI_14	2.583	1.609	0.613	− 0.881
FPI_15	3.286	1.643	0.111	− 1.122
FPI_16	2.513	1.527	0.724	− 0.625
FPI_17	3.261	1.829	0.168	− 1.371
FPI_18	3.317	1.757	0.055	− 1.323
FPI_19	2.538	1.669	0.734	− 0.784
FPI_20	3.065	1.721	0.246	− 1.205
FPI_21	2.382	1.365	0.895	0.104
FPI_22	3.367	1.664	− 0.047	− 1.197
FPI_23	1.402	0.858	2.415	5.305
FPI_26	2.090	1.450	1.096	− 0.074
FPI_27	3.271	1.882	0.028	− 1.535
FPI_29	2.854	1.433	0.425	− 0.739
FPI_30	3.327	1.480	0.310	− 0.714
FPI_31	3.477	1.534	0.119	− 1.010
FPI_32	3.960	1.769	− 0.182	− 1.410
FPI_33	3.613	1.616	0.175	− 1.213
FPI_34	4.181	1.438	− 0.342	− 0.914
FPI_35	2.980	1.392	0.580	− 0.421
FPI_36	4.161	1.485	− 0.298	− 0.949
FPI_37	3.146	1.437	0.350	− 0.713
FPI_38	3.869	1.558	− 0.274	− 1.027
FPI_39	3.126	1.592	0.209	− 1.060
FPI_40	2.322	1.309	0.777	− 0.290
FPI_41	4.121	1.572	− 0.485	− 0.832
FPI_42	3.960	1.524	− 0.330	− 0.787
FPI_43	4.070	1.384	− 0.254	− 0.816
FPI_44	4.050	1.459	− 0.403	− 0.747
FPI_45	4.975	1.361	− 1.374	1.062
FPI_46	4.211	1.472	− 0.562	− 0.524
SC	30.005	10.605	0.275	− 0.382
SeC	24.950	9.042	0.272	− 0.531
RC	18.120	7.704	0.607	− 0.261
RCL	28.553	8.837	0.058	− 0.675
NP	37.849	9.297	− 0.194	− 0.321
FPI total score	139.40	31.720	0.375	− 0.029

*SD* Standard deviation, *FPI* Fertility Problem Inventory, *SC* Social concern, *SeC* Sexual concern, *RC* Relationship concern, *RCL* Rejection of childfree lifestyle, *NP* Need for parenthood

### FPI Intercorrelations

The factor intercorrelations of the five-factor multidimensional FPI model with 43 items were calculated. The factor correlations were low to high, ranging from 0.085 (RC − RCL) to 0.704 (RCL − NP), indicating that the factors were related but distinct. The results are shown in Table 5.

**Table 5:** Correlations between the FPI dimensions and with ODSIS, OASIS and SWLS

	ODSIS	OASIS	SWLS	SC	SeC	RC	RCL
SC	0.505***	0.457***	− 0.416***				
SeC	0.486***	0.408***	− 0.433***	0.497***			
RC	0.443***	0.397***	− 0.423***	0.461***	0.636***		
RCL	0.109	0.049	− 0.142	0.173*	0.158*	0.085	
NP	0.203**	0.161*	− 0.203**	0.335***	0.234***	0.243***	0.704***

*SC* Social concern, *SeC* Sexual concern, *RC* Relationship concern, *RCL* Rejection of childfree lifestyle, *NP* Need for parenthood, *ODSIS* Overall Depression Severity and Impairment Scale, *OASIS* Overall Anxiety Severity and Impairment Scale, *SWLS* Satisfaction with Life Scale

*p < .050; **p < .010; ***p < .001

### Evidence of Construct Validity Based on Correlations with Other Variables

Regarding the relationships between the FPI dimensions and other measures (Table 5), the results showed that all the FPI dimensions had positive correlations with ODSIS and OASIS, indicating that higher scores on the FPI dimensions were associated with higher levels of depression and anxiety, providing evidence of convergent validity. The FPI dimensions also had negative correlations with SWLS, suggesting that higher scores on the FPI scale were related to lower levels of life satisfaction.

## Discussion

There is a clear need to carry out a psychosocial assessment of all women undergoing ART to better detect the emotional suffering that may arise during this process and to refer women to the most convenient psychological program. It is, therefore, essential to place emphasis on assessing the ART-related stress and the use of the FPI can facilitate this task. To the best of our knowledge, this is the first validation study of the FPI instrument in a population of Spanish women undergoing ART.

Regarding the FPI scores, our results showed that most of the items presented means above the midpoint of the scale and moderate standard deviations, which show a moderate–high level of agreement with the statements of the FPI. It should be noted that, although the dimensions showed considerable variability in their scores, the overall scores were high and showed agreement according to a normal distribution, suggesting that they are adequate.

In regard to the internal structure of the FPI, in our research, six alternative models were analyzed to examine the internal structure of the questionnaire using CFA. The unidimensional model with 46 items showed poor fit, and the results showed that the factor loadings of this model were low to moderate. This information suggests that the items did not adequately measure the single latent variable, so the model was discarded. The multidimensional model with the five dimensions suggested by the original authors of the FPI showed an acceptable fit. In line with these results, previous FPI validations have corroborated the 5-factor solutions (Kim & Shin, 2014; Peng et al., 2011; Samani et al., 2017). However, in our study, although the factor loadings of this five-dimensional model with 46 items were high, it presented three problematic items (24, 25 and 28) which had very low loadings on their respective dimension (Relationship Concerns). In the Chinese validation of the FPI (Peng et al., 2011), these items also showed some of the lowest factor loading among all items. It is possible that, in our study, these items were interpreted differently by each participant or were not properly understood due to their grammatical construction. For example, with respect to item 24 “*My partner and I could talk more openly with each other about our fertility problem/treatment*”, the expression “talk more openly” may be confusing as it is unclear if it refers to how sincerely they talk about infertility or how frequently the couple shares their fertility problems with each other. Regarding item 25 “*I couldn’t imagine us ever separating because of this*”, the ambiguous phrase “separating because of this” may cause misunderstanding. Participants may be unsure whether it refers to emotional distancing, legal separation or whether such distancing is perceived as permanent. The last discordant item is 28 “*When we talk about our fertility problem/treatment, my partner seems comforted by my comments*”. In this case, “being comforted” may be too general and clearer if specified as “my partner feels relief, supported or understood”. Finally, given that the multidimensional model with 43 items excluding these 3 items was the most appropriate, they were deleted.

Furthermore, the factorial correlations between the dimensions of the FPI were low to high, indicating that these dimensions are related, but distinct. This result is consistent with findings from the original version of the FPI (Newton et al., 1999) as well as with more recent validations in Portugal (Moura-Ramos et al., 2012) and Italy (Donarelli et al., 2015), which also showed intercorrelations among the FPI subscales ranging from 0.15 and 0.78. However, the high correlation between the domain Rejection of Childless Lifestyle (RCL) and Need for Parenting (NP) found in the Italian and Portuguese validation (Donarelli et al., 2015; Moura-Ramos et al., 2012) raises the question of whether or not these two dimensions are measuring different aspects of the same construct. In this sense, it could be argued that rejecting a childless lifestyle and having a need for parenthood are two very similar ideas, both reflecting a strong desire to have children. This idea is consistent with previous studies that proposed to unify these two dimensions into a single factor called “Importance of Parenthood” (Moura-Ramos et al., 2012). Thus, in their proposal, the FPI assesses infertility stress by evaluating two main conceptual factors: Impact on life domains (including Social Concern, Sexual Concerns and Relationship Concern) and Representations about the importance of parenthood (including Rejection of childfree lifestyle and Need for Parenthood). However, in our study, this two-factor solution did not fit well so this structure in the FPI was not supported. Further studies with more heterogeneous samples of participants would be desirable to clarify the nature and significance of these two factors and their relationship.

As in the original study (Newton et al., 1999) the present FPI validation provides strong support for its internal consistency, with high Cronbach’s alpha and McDonald’s omega coefficients reaching values up to 0.87. These results are consistent with other validations such as the Iranian version (Samani et al., 2017) and even better than those obtained in the Korean (Kim & Shin, 2014) and Italian validations (Donarelli et al., 2015). These results confirm that the FPI items in each dimension truly assess the expected construct (i.e., social concerns, sexual concerns, etc.) with good reliability outcomes.

Regarding evidence of construct validity based on the relationships between the FPI and other measures, our study shows that higher scores on the FPI dimensions are associated with higher levels of depression and anxiety as well as low scores of life satisfaction. These findings are congruent with past research (i.e., Donarelli et al., 2015; Lakatos et al., 2017; Moura-Ramos et al., 2012) and once again indicate that the experience of fertility problems and the subsequent ART may have a negative impact on the emotional wellbeing of women. In light of our results, there is no doubt that psychosocial assessments, including measures of anxiety, depression, life satisfaction and stress, should be integrated in the health services treating those women (Gameiro et al., 2015). This may prevent the chronification of psychological symptoms by facilitating the early detection of emotional distress providing the appropriate psychological intervention.

While most FPI subscales showed significant associations with anxiety, depression, and life satisfaction, supporting the construct validity of the instrument, the “Rejection of a Childfree Life” (RCL) subscale showed weaker correlations with emotional symptoms and life satisfaction. These findings are consistent with those reported by Newton et al. (1999) who also found relatively low associations between anxiety and depressive symptoms and the RCL subscale. The authors of the original scale suggested that not all domains of infertility-related stress are equally associated with emotional burden (i.e., anxiety and depressive symptoms) in infertile populations. Another possible explanation may be related with the content and formulation of the subscale items. Unlike the other FPI subscales, which ask directly about current difficulties and emotional challenges (e.g., “Holidays are especially difficult for me” or “My partner does not understand how the infertility problem affects me”), the items in the RCL subscale are formulated in the conditional or hypothetical form (e.g., “Not having a child would give me time to do other satisfying things”; “We could have a long and happy relationship without a child”) or ask about participants’ general opinions regarding life with and without children (e.g., “Couples without children are as happy as those with children”). Because these items refer to potential future scenarios rather than current experiences, their impact on participants’ present anxiety and depression may be less immediate. In contrast, the other subscales capture concrete, day-to-day impacts of infertility (Newton et al., 1999), which may explain their stronger associations with measures of psychological distress. However, these explanations are tentative, and we cannot draw robust in this direction, future research with more targeted measures and larger samples is needed to explore this issue in greater depth.

These findings point out that the FPI is an instrument that demonstrates adequate psychometric properties for the intended purpose and the sample used, suggesting that it could be useful for Spanish populations and its potential to facilitate the clinical practice of professionals in medical settings. However, there are several limitations that must be considered. First, the sample was comprised only of Spanish heterosexual women. In this sense, the cultural characteristics of Spanish women (gender stereotypes, types of families, expectations about motherhood, etc.) should also be considered, as they may affect the answers to the items. It should also be noted that it was not possible to include in the sample single or LGBTQI+ women as some items cannot be answered or make no sense for such populations (i.e., “I find I’ve lost my enjoyment of sex because of fertility problem” or “My partner and I work well together handling questions about our infertility”). Future efforts should be conducted to determine whether relationship and sexual dimensions of the FPI can be modified to be more inclusive or if some items should be removed to obtain an instrument more representative of current family structures. Additionally, the FPI does not provide a clinical cut-off point that could help determine when clinical symptoms emerge, which may facilitate decision-making regarding how and when psychological intervention should be provided. Also importantly, it is important to note that comparisons with other studies are limited, as there are many methodological differences between them (e.g. characteristics of the study population, type of statistical analyses, number of factors obtained). Finally, the choice of a CFA approach should be interpreted in light of sample size considerations, as the relatively small sample (n = 199), in relation to the large number of items, represents a limitation that may affect the stability of the estimated model.

Despite these limitations, this study provides a Spanish version of the FPI with adequate psychometric properties (reliability and construct validity) that could contribute to improve both research and clinical practice in health services that provide ART. The validation of the FPI Spanish version may facilitate the assessment of fertility-related stress in all women receiving ART and support or refer them to the most convenient service when significant stress is detected. This may help both stakeholders, health professionals, to provide integral care, and women, who could receive higher quality care. Although further analyses with more heterogeneous samples (i.e., men, or women with diverse marital status) are needed, this study provides new evidence on the FPI structure and a Spanish validation of the instrument with 43-item and five-factor model that ensures adequate assessment of ART-related stress.

## Supplementary Information

Below is the link to the electronic supplementary material.

Supplementary file1 (DOCX 18 kb)

## Data Availability

Data are available from the authors upon reasonable request to the corresponding author.
