# Supplementary material for: Fertility Problem Inventory: Psychometric Properties and Validation in Spanish Women Undergoing Assisted Reproduction Treatment
Source: J Clin Psychol Med Settings. 2026 Feb 12;33(3):545–55. doi: 10.1007/s10880-026-10131-6 (PMC13582040; doi:10.1007/s10880-026-10131-6)
Supplement: Supplementary file 1 [file 10880_2026_10131_MOESM1_ESM.docx]

**Appendix A.**

**Fertility Problem Inventory (FPI) - Spanish version**

*Newton et al., (1999) traducido por G. Lasheras and G. Mestres (Hospital Universitari Dexeus). Validación española por Martínez-Borba et al., (2025)*

Este cuestionario ha sido diseñado para medir el malestar emocional, creencias y actitudes relacionadas con la infertilidad. Por favor, responde con la mayor precisión posible. Elige la respuesta que te parezca más adecuada.

| Totalmente en desacuerdo | En desacuerdo | Algo en desacuerdo | Algo de acuerdo | De acuerdo | Totalmente de acuerdo |
| --- | --- | --- | --- | --- | --- |
| 1 | 2 | 3 | 4 | 5 | 6 |

| 1. **No me molesta cuando me hacen preguntas acerca de niños/as.** |  |
| --- | --- |
| 2. **No parece que los miembros de la familia nos traten de forma diferente.** |  |
| 3. Los días festivos son especialmente difíciles para mí. |  |
| 4. Las reuniones familiares son especialmente difíciles para mí. |  |
| 5. No puedo dejar de compararme con amistades que tienen hijos/as. |  |
| 6. **Todavía tengo muchas cosas en común con amistades que tienen hijos/as.** |  |
| 7. Me resulta difícil pasar tiempo con amistades que tienen hijos/as pequeños/as. |  |
| 8. Cuando veo a familias con niños/as me siento excluida/o. |  |
| 9. Siento como si amistades o familiares nos dieran de lado. |  |
| 10. **No me molesta cuando los demás hablan de sus hijos/as.** |  |
| 11. Disfruto menos del sexo por el problema / tratamiento de fertilidad. |  |
| 12. **Me siento igual de atractiva/o para mi pareja que antes.** |  |
| 13. **No me siento diferente a otras personas de mi mismo sexo.** |  |
| 14. Siento que he fracasado en el sexo. |  |
| 15. Durante las relaciones sexuales, lo único que puedo pensar es en querer tener un/a hijo/a (u otro/a hijo/a). |  |
| 16. Tener relaciones sexuales me resulta difícil porque no quiero otra decepción. |  |
| 17. Puedo sentirme muy enfadada/o si perdemos un día fértil para tener relaciones sexuales. |  |
| 18. A veces siento tanta presión que tener relaciones sexuales se hace difícil. |  |
| 19. No puedo mostrarle a mi pareja cómo me siento porque esto hará que se disguste. |  |
| 20. Mi pareja no entiende de qué manera me afecta el problema / tratamiento de fertilidad. |  |
| 21. **Mi pareja y yo resolvemos bien cuestiones sobre nuestra infertilidad (o sobre los tratamientos de reproducción).** |  |
| 22. Me molesta que mi pareja reaccione de manera diferente frente al problema / tratamiento de fertilidad. |  |
| 23. Mi pareja está bastante decepcionada/o conmigo. |  |
| 24. *Podríamos hablar más abiertamente entre nosotros/as acerca de nuestro problema / tratamiento de fertilidad. |  |
| 25. ***No puedo imaginar que nos separemos debido a esto.** |  |
| 26. Cuando intentamos hablar de nuestro problema / tratamiento de fertilidad, acabamos discutiendo. |  |
| 27. Me preocupa que nos distanciemos debido al problema / tratamiento de fertilidad. |  |
| 28. ***Cuando hablamos de nuestro problema / tratamiento de fertilidad, parece que mi pareja se consuela con mis comentarios.** |  |
| 29. **Las parejas sin hijos/as son tan felices como las que tienen hijos/as.** |  |
| 30. **Puedo verle una serie de ventajas al hecho de no tener un/a hijo/a (u otro/a hijo/a).** |  |
| 31. **Puedo visualizar una vida feliz juntos/as, sin un/a hijo/a (u otro/a hijo/a).** |  |
| 32. **A veces me pregunto seriamente si quiero un/a hijo/a (u otro/a hijo/a).** |  |
| 33. **No tener un/a hijo/a (u otro/a hijo/a) me aportaría tiempo para hacer otras cosas satisfactorias.** |  |
| 34. **Para mi felicidad no es necesario tener un/a hijo/a (u otro/a hijo/a).** |  |
| 35. **Podríamos tener una relación larga y feliz sin un/a hijo/a (u otro/a hijo/a).** |  |
| 36. **No tener hijos/as conlleva una cierta libertad que me atrae.** |  |
| 37. El embarazo y el parto son los dos acontecimientos más importantes en la relación de pareja. |  |
| 38. Para mí, ser madre/padre es un objetivo más importante que tener una carrera satisfactoria. |  |
| 39. Mi matrimonio (o relación de pareja) necesita un/a hijo/a (u otro/a hijo/a). |  |
| 40. Es difícil sentirse como una persona adulta hasta que no se tiene un/a hijo/a. |  |
| 41. Me asusta pensar en un futuro sin un/a hijo/a (u otro/a hijo/a). |  |
| 42. Me siento vacía/o debido a los problemas / tratamientos de fertilidad. |  |
| 43. **Tener un/a hijo/a (u otro/a hijo/a) no es el objetivo principal de mi vida.** |  |
| 44. A menudo siento que yo he nacido para ser madre/padre. |  |
| 45. Siempre he querido ser madre/padre. |  |
| 46. Haré cualquier cosa para tener un/a hijo/a (u otro/a hijo/a). |  |

**Nota*: en la validación al español de Martínez-Borba et al., 2025 se propone eliminar los ítems 24, 25 y 28.

Los ítems marcados en negrita son inversos y se debe invertir la puntuación.
